# Influences of Smartphone and Computer Use on Health-Related Quality of Life of Early Adolescents

**DOI:** 10.3390/ijerph19042100

**Published:** 2022-02-13

**Authors:** Mei-chun Cheung, Janelle S. K. Lai, Joanne Yip

**Affiliations:** 1 Department of Social Work, The Chinese University of Hong Kong, Hong Kong SAR, China; janellelsk@gmail.com; 2 Institute of Textiles and Clothing, The Hong Kong Polytechnic University, Hong Kong SAR, China; joanne.yip@polyu.edu.hk

**Keywords:** smartphone, computer, health-related quality of life, early adolescents

## Abstract

This study explored the daily amount of time that early adolescents spent using smartphones and computers, and their influences on health-related quality of life of early adolescents. A total of 650 early adolescents were recruited. The 36-Item Short Form Health Survey was used to measure their health-related quality of life. The early adolescents reported their average daily time spent using smartphones and computers over the course of the previous week; the majority of early adolescents (71%) spent approximately 1 h a day or less using computers on average or reported no computer use, and 98.8% indicated that they used smartphones for less than 1 h to more than 4 h per day on average. The results showed that the average daily time spent using smartphones was significantly negatively associated with two scales in the physical domain and four scales in the mental domain of health-related quality of life of early adolescents, whereas the average daily time spent using computers was significantly negatively associated with two scales in the mental domain (*p* < 0.05). Therefore, early adolescents who spent more time using smartphones and computers have significantly poorer outcomes in the physical and mental domains of their health-related quality of life.

## 1. Introduction

Smartphones and computers have become the most frequently used technological devices, and they are now widely regarded as necessities in the lives of young people. With multiple functions due to different social media and technology being combined into one device, smartphones and computers have brought convenience and efficiency to many people [1]. The integration of computer technology into the learning experience extends students’ access to various resources and information and allows them to learn and develop skills independently via computers [2], suggesting the beneficial effects of computer technology on the quality of life of adolescents. Students studying in the 7th to 12th grades with computer access at home report better overall grades in both mathematics and English than students without such access [3]. Smartphones allow adolescents to understand their environment, connect with their peers through social media, and access entertainment. It is also suggested that smartphones can enhance the autonomy of adolescents as the devices enable adolescents to experience life independently from their parents [4]. Some studies have even supported that the internet provides middle/late adolescents and adults with a chance to speak out, have their thoughts heard and validated, and build better relationships [5,6,7].

Despite smartphones and computers having the benefits of convenience, flexibility, and multifunctionality, it was found that approximately 40% of secondary school students used electronic screen devices for 1 to 3 h per day and that 23% used them for more than 3 h per day [8]. In Hong Kong, while the majority of adolescents spent less than or approximately 1 h per day using computers, approximately 83% of 390 early adolescent girls aged 11 to 14 spent 1 to 3 h per day using a smartphone [9]. Therefore, the negative effects of prolonged/extensive smartphone and/or computer use on physical and psychosocial outcomes among adolescents have been highlighted for investigation in view of the popularity of these devices among adolescents. For instance, prolonged time spent focusing on smartphone and computer screens was found to cause multiple physical symptoms related to poor ocular health, such as eye strain and eye fatigue, among middle adolescents aged 14 to 18 [10]. Nearly 45% of adolescents (aged 10 to 19) using smart devices reported eye discomfort [11]. Adolescents who use smartphones or computers for 2 h or more per day on average are more prone to bodily pain and musculoskeletal discomfort, such as neck, shoulder, wrist, finger, and lower back pain [1,12,13,14], which may be the consequence of physical inactivity and poor posture [1]. For adolescents who use a computer for more than 2 h per day, the associated head and lower back pain may cause moderate or severe inconvenience in their everyday lives [12]. Longer durations of smartphone and computer use in bed are associated with adolescent sleep problems [15], including poorer sleeping patterns, shorter sleep duration, worse sleep quality, and excessive daytime sleepiness [16,17,18,19,20]. Psychosocially, more time spent on smartphones and computers is associated with lower psychological well-being and less emotional stability [21]. Adolescents who use smartphones and computers excessively also have a higher likelihood of showing symptoms of anxiety and depression [13,21,22]. In addition, extensive smartphone and computer use can have negative consequences for cognitive control, executive ability, and productivity in adolescents, leading to poorer academic performance [21,23,24]. Regarding the social dimension, relationships with parents and friends may be adversely affected by conflicts arising from smartphone use [1,24] and by the physical disconnection and lack of copresence associated with heavy use of mobile phones [25]. More time spent on smartphones and computers by adolescents has also been found to be associated with increased difficulty making friends and being cared for [21].

While most previous studies have usually included middle and/or late adolescents in their study samples [3,4,6,10,13,14,18,19,20,22], the influences of smartphone and computer use, particularly on health-related quality of life of early adolescents, has been overlooked, as smartphones and computers have not commonly been used among early adolescents in the past. However, as part of the rising general popularity of smartphones and computers, there has recently been a substantial increase in the number of smartphone and computer users among the early adolescent population. For instance, in Hong Kong, the percentage of early adolescents who own a smartphone has almost doubled, from 46.1% in 2012 to 81.3% in 2019 [26,27]. In 2019, every early adolescent surveyed in a study in Hong Kong reported having used a personal computer over the past 12 months [27]. As the number of early adolescents owning smartphones and/or computers has dramatically increased in recent years, it is essential to explore the influences of smartphone and computer use on their health-related quality of life to determine whether smartphone and computer use displaces social activities, leading to social withdrawal and a decline in well-being (in line with the displacing social activity hypothesis), or whether it enables the development of additional social ties established on the internet (in line with the displacing strong ties hypothesis), as proposed by Kraut et al. [28]. To fill this research gap, this study explored the prevalence of smartphone and computer use among early adolescents in Hong Kong and the influences of smartphones and computers on their health-related quality of life. Specifically, this study aimed to investigate (1) the amount of time spent using a smartphone and/or computer among early adolescents in Hong Kong and (2) their influences on the physical and mental domains of health-related quality of life of early adolescents. Based on previous studies on middle/late adolescents, [10,13,14,18,19,20,22], it was hypothesized that similar to middle/late adolescents, the amount of time that early adolescents spent using smartphones and/or computers would negatively affect the physical and mental domains of health-related quality of life of early adolescents, in line with the displacing social activity hypothesis proposed by Kraut et al. [28].

## 2. Materials and Methods

### 2.1. Participants

A cross-sectional study was conducted using self-report questionnaires in Hong Kong from 2018 to 2019. A total of 650 early adolescents aged 11 to 13 were recruited from five secondary schools, which involved a convenient sampling of one co-ed school and four all-girls schools in Hong Kong that had been collaborating in a school screening program jointly conducted by The Hong Kong Polytechnic University and The Chinese University of Hong Kong for spine deformity since 2012. As a result, the study sample consisted of 569 females (87.5%) and 81 males (12.5%). Recruitment was conducted through the secondary schools and carried out at the secondary schools during weekends. Upper body posture screening was provided to the early adolescents as an incentive. Early adolescents anonymously filled in the self-report questionnaires individually within the sight of their parents or guardians, who sat in the waiting area located nearby. After completion, the early adolescents submitted their self-report questionnaires to the research assistants before undergoing posture screening, and the questionnaires were double-checked by the research assistants to avoid multiple submissions. All of the early adolescents took part voluntarily, and informed assent and written informed consent for participation were obtained from early adolescents and their parents or guardians, respectively, prior to the study. The study was conducted according to the guidelines of the Declaration of Helsinki, and the research protocol was approved by the Human Subjects Ethics Sub-committee of The Hong Kong Polytechnic University, the Survey and Behavioral Research Ethics Committee, and the Joint Chinese University of Hong Kong—New Territories East Cluster Clinical Research Ethics Committee of The Chinese University of Hong Kong. All methods were performed in accordance with the relevant guidelines and regulations. The demographic information and descriptive statistics on the main variables of interest in early adolescents are provided in Table 1.

### 2.2. Measures

#### 2.2.1. Health-Related Quality of Life

Early adolescents’ health-related quality of life in the physical and mental domains was measured by the Hong Kong version [29,30,31,32] of the 36-item Short Form Health Survey (SF-36) [33,34], which has been adapted and validated for over 40 populations [35] and is commonly used in adolescents [36,37,38], with norm references available from 14 populations [35], including Hong Kong [29,30]. The SF-36 consists of 35 questions that are summarized into eight multi-item scales under two domains and one self-reported question related to changes in health compared to one year ago. The four scales related to the physical domain of health-related quality of life are physical functioning (10 items), role limitation due to physical health problems (4 items), bodily pain (2 items), and general health (5 items), whereas the four scales related to the mental domain of health-related quality of life are vitality (energy/fatigue; 4 items), social functioning (2 items), role limitation due to emotional problems (3 items), and mental health (5 items) [39]. The items in each scale are summed and transformed according to the standard scoring algorithm of the SF-36 [34] into a standardized scale score for each scale that ranges from zero to 100, with a higher scale score indicating better health-related quality of life. As this study does not focus on changes in health over time, the self-reported health transition item, which is an independent question that is not used to score any of the eight multi-item scales [34], was not used for analysis in this study.

#### 2.2.2. Smartphone and Computer Use

A custom questionnaire was used to measure the amount of time that early adolescents spent using smartphones and computers. The respondents were asked to rate their average daily time spent using computers or smartphones over the previous week on the following scale: (a) never, (b) less than one hour, (c) one to two hours, (d) three to four hours, and (e) more than four hours. The scales for the average daily time spent performing these activities were adapted from the study by Kratěnová et al. [40], where it was determined that early adolescents spent an average of 2 h per day using the computer. The questionnaire has been used in our previous studies [9,41], where the scales generally achieved a normal distribution for the data being collected.

### 2.3. Data Processing and Analysis

All statistical analyses were performed using the Statistical Package for Social Sciences (SPSS, Version 25.0, IBM Corp, Armonk, NY, USA). Descriptive statistics were generated for the total sample and each gender/age group sub-sample. Quantitative measurements are summarized as the mean ± standard deviation and *n* (%). The comparison of the amount of time spent by early adolescents using smartphones and computers was performed with a paired-sample *t*-test, and comparisons between males and females and different age groups on the amount of time spent using smartphones and computers were performed with Welch independent sample *t*-tests or analysis of variance (ANOVA) followed by nonparametric Mann–Whitney U tests to account for the unequal sample sizes. Bivariate Pearson’s correlations were used to measure the relationship between the average daily amount of time spent using smartphones and computers and the physical and mental domains of health-related quality of life. Stepwise multiple linear regressions were conducted to assess whether the average daily amount of time spent by early adolescents using smartphones and/or computers was a significant predictor of each of the eight scales related to the physical and mental domains of health-related quality of life after controlling for confounding effects. The covariates that were found to be associated with the average daily time spent using smartphones and/or computers at *p <* 0.05 in the bivariate analyses were entered into the models. Preliminary analyses were conducted to ensure no violation of the assumptions of normality, linearity, multicollinearity, or homoscedasticity. A *p*-value of less than 0.05 was considered statistically significant.

## 3. Results

### 3.1. The Prevalence of Smartphone and Computer Use

**Figure 1:**
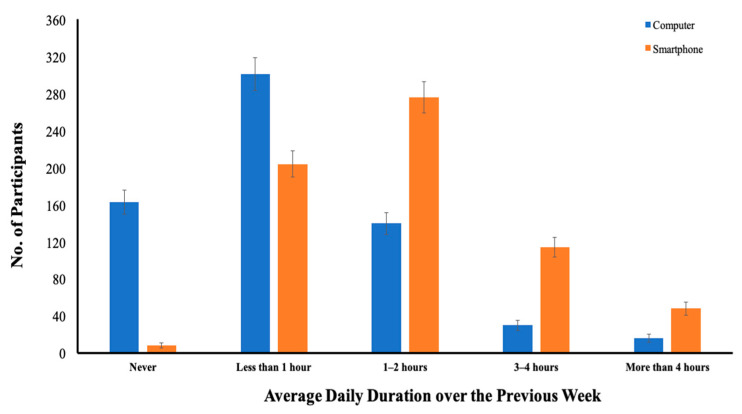
Distribution of early adolescents categorized based on their average daily time spent using a smartphone or computer over the previous week (*n* = 650).

Figure 1 shows the numbers of early adolescents categorized based on their average daily time spent using a smartphone or computer over the previous week. While 8 of the adolescents reported no smartphone usage over the previous week, the majority of early adolescents (98.8%) were smartphone users. On average, 42.5% of the early adolescents used their smartphones for one to two hours per day, 31.4% used their smartphones for less than one hour per day, 17.5% used their smartphones for three to four hours per day, and 7.4% used their smartphones for more than four hours per day. A total of 487 early adolescents had used a computer over the previous week, accounting for nearly 74.9% of the sample. On average, 46.3% used computers for less than one hour per day, 21.5% used computers for one to two hours per day, 7.1% used computers for three to four hours or more per day, and 25.1% reported no computer usage over the previous week.

Apart from the greater popularity of smartphones among early adolescents, the results of the paired-sample *t*-test suggested that on average, the early adolescents spent more time daily using smartphones than they did using computers over the course of the previous week, *t*(649) = 18.725, *p* < 0.001. Welch independent sample *t*-tests were conducted to compare any differences between males and females in their average daily time spent using computers. The results showed that males spent more time using computers than females, *t*(1, 97.07) = 2.026, *p* < 0.05. The nonparametric Mann–Whitney U test demonstrated similar significant results between males (mean rank: 361.43) and females (mean rank: 320.39), *U* = 20134.5, *z* = -1.968, *p* < 0.05. No significant difference between males (mean rank: 291.44) and females (mean rank: 330.35) was found in their average daily time spent using smartphones over the previous week, in either the parametric, *t*(1, 97.63) = −1.250, *p* > 0.05, or nonparametric tests, *U* = 20285.5, *z* = −1.853, *p* > 0.05. The distribution of the average daily time spent by males and females using smartphones and computers and the test statistics are presented in Table 2. Regarding the effects of age, no significant differences were found in the average daily time spent using a smartphone or computer over the previous week after adjusting for unequal sample size, *p* > 0.05, in either the parametric or nonparametric tests.

### 3.2. Correlations between Smartphone and Computer Usage and Early Adolescents’ Health-Related Quality of Life

Pearson’s correlations were used to reveal the relationships between the average daily time spent by early adolescents using smartphones or computers over the previous week and the eight scales related to the physical and mental domains of health-related quality of life. The correlations are presented in Table 3. The results indicated that the average daily time spent using smartphones over the previous week was significantly negatively correlated with two scales related to the physical domain of health-related quality of life: (i) bodily pain, *r*(650) = −0.108, *p* < 0.01, and (iii) general health, *r*(650) = −0.124, *p* < 0.01. In addition, the average daily time early spent by early adolescents using smartphones over the previous week was significantly negatively correlated with all four scales related to the mental domain of health-related quality of life: (i) vitality (energy/fatigue), *r*(650) = −0.180, *p* < 0.001; (ii) social functioning, *r*(650) = −0.146, *p* < 0.001; (iii) role limitation due to emotional problems, *r*(650) = −0.124, *p* < 0.01; and (iv) mental health, *r*(650) = −0.201, *p* < 0.001. However, no significant relationship between the average daily time spent by early adolescents using smartphones over the previous week and the physical functioning or role limitation due to physical health problems scales was observed.

Regarding the use of computer, the average daily time early adolescents spent using computers over the previous week was significantly negatively correlated with one scale related to the physical domain of health-related quality of life, namely general health, *r*(650) = −0.093, *p* < 0.05, and three scales related to the mental domain of health-related quality of life: (i) vitality (energy/fatigue), *r*(650) = −0.130, *p* < 0.001; (ii) social functioning, *r*(650) = −0.081, *p* < 0.05; and (iii) mental health, *r*(650) = −0.124, *p* < 0.01. However, no significant relationship between the average daily time early adolescents spent using computers over the previous week and the scales for physical functioning, role limitation due to physical health problems, bodily pain, or role limitation due to emotional problems was observed.

### 3.3. Influences of the Average Daily Time Spent Using Smartphones and Computers on Health-Related Quality of Life of Early Adolescents

Given that the average daily time spent by early adolescents using smartphones and computers were significantly correlated with each other, *r*(650) = −0.201, *p* < 0.001, stepwise multiple linear regressions were conducted to predict the associated physical and mental domains of health-related quality of life of early adolescents based on their average amount of time spent using smartphones and computers per day. The results are presented in Table 4.

In general, the average amount of time spent by early adolescents using smartphones per day was the first significant predictor entered into the regression models. For the physical domain of health-related quality of life, the average amount of time spent using smartphones per day statistically significantly predicted 1.2% of the variance in bodily pain, *F* (1, 648) = 7.691, *p* < 0.01, *R*^2^ = 0.012, and 1.5% of the variance in general health, *F* (1, 648) = 10.137, *p* < 0.01, *R*^2^ = 0.015. The average daily amount of time spent by early adolescents using smartphones negatively affected bodily pain (*b* = −1.515, *p* < 0.01) and general health (*b* = −2.372, *p* < 0.01). For the mental domain of quality of life, the average daily amount of time spent using smartphones also statistically significantly predicted 3.2% of the variance in vitality (energy/fatigue), *F* (1, 648) = 21.717, *p* < 0.001, *R*^2^ = 0.032; 2.1% of the variance in social functioning, *F* (1, 648) = 14.151, *p* < 0.001, *R*^2^ = 0.021; 1.5% of the variance in role limitation due to emotional problems, *F* (1, 648) = 10.118, *p* < .01, *R*^2^ = 0.015; and 4.1% of the variance in mental health, *F* (1, 648) = 27.395, *p* < 0.001, *R*^2^ = 0.041. The average daily amount of time spent by early adolescents using smartphones negatively affected vitality (energy/fatigue) (*b* = −3.432, *p* < 0.001), social functioning (*b* = −2.063, *p* < 0.001), role limitation due to emotional problems (*b* = −3.209, *p* < 0.01), and mental health (*b* = −3.670, *p* < 0.001). That is, early adolescents who spent more time using their smartphones per day on average had poorer health-related quality of life in terms of bodily pain, general health, vitality (energy/fatigue), social functioning, role limitation due to emotional problems, and mental health.

After the effects of the average daily amount of time spent by early adolescents using smartphones were controlled, the average amount of time spent using computers per day was entered into the regression models and still significantly predicted two scales related to the mental domain of health-related quality of life, namely vitality (energy/fatigue) and mental health. The average daily amount of time spent by early adolescents using computers statistically significantly predicted an additional 0.9% of the variance in vitality (energy/fatigue), *F* (1, 647) = 6.226, *p* < 0.05, *R*^2^ change = 0.009, and an additional 0.7% of the variance in mental health, *F* (1, 647) = 4.998, *p* < 0.05, *R*^2^ change = 0.007. The average daily time spent using computer also negatively affected vitality (energy/fatigue) (*b* = −1.845, *p* < 0.05) and mental health (*b* = −1.575, *p* < 0.05). That is, early adolescents who spent more time per day using a computer on average had poorer health-related quality of life in terms of vitality (energy/fatigue) and mental health. As a result, both the average daily time spent using smartphones and computers significantly predicted the variance in vitality (energy/fatigue) and mental health. The two predictors significantly predicted 4.2% of the variance in vitality (energy/fatigue), *F* (2, 647) = 14.059, *p* < 0.001, *R*^2^ = 0.042, and 4.8% of the variance in mental health, *F* (2, 647) = 16.281, *p* < 0.001, *R*^2^ = 0.048.

## 4. Discussion

This study aimed to investigate the average daily amount of time spent by early adolescents using smartphones and computers and their influences on the physical and mental domains of health-related quality of life of early adolescents in Hong Kong. The results revealed that early adolescents use smartphones more than computers and that they spent more time on their smartphones than they did on their computers. While 98.8% of the early adolescents in the present study were smartphone users, only 74.9% were computer users. The findings were similar to the rates of ownership of computers and smartphones for people aged 10 and above in Hong Kong reported in 2019, with 77.6% owning computers and 91.5% owning smartphones [27]. In addition, while nearly half of the early adolescents used computers for less than an hour per day, substantially more adolescents used their smartphones than their computers for an average daily duration of one hour or more. Most early adolescents used their smartphones for one to two hours a day, which was nearly double the number of early adolescents who used computers for the same duration. The number of early adolescents who used their smartphones for three to four hours or more was nearly three times the number of early adolescents who used computers for the same amount of time. The findings further established the rising popularity of smartphones, which are recognized for their benefits of convenience, flexibility, and multifunctionality, for daily use among early adolescents.

The influences due to the average daily amount of time spent by early adolescents using smartphones and computers on health-related quality of life differed. Our results suggested longer duration of using a smartphone was associated with poorer health-related quality of life in two scales of the physical domain, that is, general health and bodily pain, and in all four scales related to the mental domain: vitality (energy/fatigue), social functioning, role limitation due to emotional problems, and mental health (*p* < 0.05). In contrast, higher amounts of time spent using a computer were associated with poorer health-related quality of life on only two scales related to the mental domain, that is, the vitality (energy/fatigue) and mental health scales (*p* < 0.05). Although the average daily time spent using smartphones and computer use were not negatively associated with all of the scales included in the physical and mental domains of health-related quality of life of early adolescents, which was the expectation, the negative effects of the higher amounts of time spent using smartphone on health-related quality of life of early adolescents were more significant, adversely influencing a total of two of the scales related to the physical domain and all four scales related to the mental domain of health-related quality of life, than those affected by the amount of time spent using a computer, which only affected two scales related to the mental domain of health-related quality of life. In addition, the findings were in line with the understanding that average daily screen time that was longer than one hour was associated with poorer physical and mental wellbeing, especially among early adolescents [21,42]. Moreover, these findings are generally more in line with the displacing social activity hypothesis than the displacing strong ties hypothesis as proposed by Kraut et al. [28], as the results revealed significant more time spent using a computer or smartphone had an adverse influence on early adolescents’ health-related quality of life, as revealed by significant negative associations with various scales of SF-36. This finding is particularly concerning because early adolescents at this life stage may be especially vulnerable to the negative impact of prolonged smartphone and computer use, as they are undergoing physiological, psychological, and social changes, and their lifestyle patterns and personality are developing [11,43,44]. These life changes and the induction of stress may be accompanied by social and play behaviors that are associated with risk-taking, novelty, exploration, and sensation-seeking and that may contribute to computer and smartphone addiction [45], which is associated with physical and psychosocial issues that cause significant harm to various aspects of an addict’s life, well-being, and quality of life [24,43,46,47,48]. Prolonged/extensive computer and smartphone use is also positively associated with suicide-related outcomes, such as self-injurious behavior and suicide attempts [23,24,49,50]. Future studies can investigate the prevalence of computer and smartphone addiction among early adolescents, especially during COVID-19, to further investigate the negative impact of the dependence/addictive use of smartphones and computers.

Surprisingly, compared to the amount of time spent using a smartphone, a higher amount of time spent using computers contributed less to both mental health and vitality (energy/fatigue) in the regression models. In addition, it did not predict any scale of the physical domain of health-related quality of life, which was contrary to our hypothesis and the findings of previous studies that indicated that prolonged adolescent computer use and smartphone use are associated with poorer ocular health [10], bodily pain, and musculoskeletal discomfort [1,12,13,14] as well as poorer sleep patterns and quality [16,17,18,19,20]. As advancements in technology have resulted in smartphones having bigger screens, more functions, enhanced performance, and internet connectivity [51], some functions and activities that were originally performed using computers may be shifted to smartphones. For example, more people are shifting away from playing computer and console games to playing mobile games [52], and there has also been an increase in the use of smartphones for communication, watching videos and video sharing, small tasks, and information searches [51,52,53]. While nearly all of the early adolescents in the present study were smartphone users, approximately 71% of the participants had not used a computer (25.1%) or used a computer for less than an hour per day (46.3%) over the previous week. This difference in the time spent using smartphones versus computers may suggest the difference in the functions of a smartphone and computer and the activities being performed by early adolescents, which may further explain the discrepancy between the extent of the adverse influence due to amounts of time spent using computers or smartphones on health-related quality of life. Indeed, longer duration in using a smartphone leads to smartphone dependence [52,54] and causes a negative impact on social and academic behavior, wellbeing, and quality of life in adolescents [55,56]. Therefore, the findings on the discrepancy between the average daily time spent using smartphones and computers, and the variation in their influences on health-related quality of life in our study substantiate the notion that dependence on or addiction to smartphones and/or computers and their negative impact on health-related quality of life of adolescents may be minimized when these technologies are used within healthy limits, which highlights the importance of further investigating the optimal amount of time spent by early adolescents using smartphones and computers to lessen any adverse influences on their health-related quality of life. Although no conclusive recommendations have been made for early adolescents regarding screen time, a systematic review showed that more than two hours of screen time per day was significantly associated with a variety of negative health outcomes and lower quality of life [57].

This study deepens our understanding of the prevalence of computer and smartphone use among early adolescents in Hong Kong and its influence on the physical and mental domains of health-related quality of life of early adolescents. Nonetheless, the significance of the study should be considered along with the following limitations. First, due to convenient sampling, the majority of the participants were female (87.5%) and 12 years old (67.1%). This may reduce the generalizability of the current findings and may reflect the situation of early female adolescents aged 12 years to a larger extent. In fact, early adolescents experience rapid growth and changes both internally and externally during puberty [58], and such changes may affect why and how they use technology as well as the amount of time that they spend using it. As girls and boys experience puberty and adolescence differently, prolonged smartphone use and its negative effects may be more prominent among girls. Billieux et al. [59] found that adolescent girls had a higher likelihood of dependence on and addiction to smartphones. Adolescent girls were also reported to have more severe health problems than adolescent boys [13] and to be more vulnerable to the negative effects of prolonged smartphone use [60]. Regarding the effects of age, given the narrow age range in our study, that is, from 11 to 13 years old, the developmental differences in the amount of time spent using smartphones and computers were not obvious, but developmental impacts may be possible if adolescents within a wider age range can be examined. Therefore, further studies can investigate and examine gender differences and the effects of age in terms of time spent using smartphones and computers, and their influence on health-related quality of life of early adolescents. Second, the results were based on self-report measures of smartphone and computer usage. In particular, the average daily amount of time spent using a computer and smartphone was measured subjectively, and there may be discrepancies between self-reported usage and actual usage [61]. The different kinds of computer and smartphone activities being performed by adolescents were also not captured and analyzed. In future research, log data on the activities performed by early adolescents when they used their smartphones and/or computers should be collected, if possible, to provide more comprehensive, objective observations on both the amount of time spent by early adolescents and types of activities being performed. Third, although there was no significant difference in the average daily time spent using a smartphone between males and females, early adolescent boys spent more time on computers than early adolescent girls did. Given that the sample size of early adolescent boys was relatively small compared to that of early adolescent girls, it would be worthwhile to recruit more early adolescent boys in the future to explore whether there are any differential effects of the time spent using smartphones and computers on the different genders. Finally, this study was a cross-sectional study and captured the influences of the time spent using computers and smartphones on health-related quality of life of early adolescents at only one time-point. Longitudinal studies can be conducted in the future to capture variation in the influences of these variables throughout adolescence.

## 5. Conclusions

This study filled a knowledge gap by exploring the average daily amount of time that early adolescents spent using smartphones and computers and their influences on the physical and mental domains of health-related quality of life of early adolescents. The empirical evidence suggested that early adolescents spent more time using smartphones than they did using computers and that higher amounts of time spent using a smartphone and computer resulted in significantly poorer physical and mental domains of health-related quality of life of early adolescents. As early adolescents may have increased the amount of time spent using computers or smartphones due to the COVID-19 pandemic, the findings further highlight the importance of identifying the optimal amount of time spent by an early adolescent using a smartphone and/or computer to minimize any negative effects on their health-related quality of life.

## Figures and Tables

**Table 1:** Demographic information and descriptive statistics on SF-36 scales.

Variable	N (%)	Mean	SD	Range
Age (years)	650	11.902	0.566	11–13
11	139 (21.4)			
12	436 (67.1)			
13	75 (11.5)			
Sex				
Male	81 (12.5)			
Female	569 (87.5)			
SF-36 Scales				
Physical functioning	650	94.454	15.072	0–100
Role limitation due to physical problems	650	96.346	14.191	0–100
Bodily pain	650	91.928	12.779	41–100
General health	650	77.063	17.465	15–100
Vitality (energy/fatigue)	650	73.031	17.415	15–100
Social functioning	650	91.942	12.897	25–100
Role limitation due to emotional problems	650	89.436	23.650	0–100
Mental health	650	74.363	16.649	8–100

SF-36: 36-item Short Form Health Survey.

**Table 2:** Descriptive and inferential statistics for the average daily time spent using smartphones and computers between males (*n* = 81) and females (*n* = 569).

Average Daily Time Spent	Smartphone		Computer	
	Males (*n* = 81)	Females (*n* = 569)	Males (*n* = 81)	Females (*n* = 569)
Never	2	6	17	146
Less than 1 h	36	168	33	268
1–2 h	23	253	21	119
3–4 h	12	102	8	22
More than 4 h	8	40	2	14
**Test Statistic**	***t/z***	***p***	***t/z***	***p***
Welch test	−1.250	0.162	2.026	0.045
Mann–Whitney U test	−1.853	0.064	−1.968	0.048

**Table 3:** Correlations between the average daily time spent by early adolescents using smartphones and computers over the previous week and the scales of SF-36 (*n* = 650).

Scales of SF-36	Smartphone Usage	Computer Usage
Physical functioning	0.017	−0.024
Role limitation due to physical health problems	−0.064	−0.022
Bodily pain	−0.108 **	−0.059
General health	−0.124 **	−0.093 *
Vitality (energy/fatigue)	−0.180 ***	−0.130 ***
Social functioning	−0.146 ***	−0.081 *
Role limitation due to emotional problems	−0.124 **	−0.064
Mental health	−0.201 ***	−0.124 **

SF-36: 36-item Short Form Health Survey. * *p* < 0.05 (two-tailed). ** *p* < 0.01 (two-tailed). *** *p* < 0.001 (two-tailed).

**Table 4:** Summary of the stepwise multiple linear regressions of the average daily time spent by early adolescents using smartphones and computers as predictors of the physical and mental domains of SF-36 (*n* = 650).

Scales of SF-36	Model 1: Smartphone Use				Model 2: Computer Use			
	*R* ^2^	*F* Value	*p* Value	*β*	*R*^2^∆	*F* Value ∆	*p* Value	*β*
Bodily pain	1.2%	7.691	0.006 **	−1.515				
General health	1.5%	10.137	0.002 **	−2.372				
Vitality (energy/fatigue)	3.2%	21.717	0.000 ***	−3.432	0.9%	6.226	0.013 *	−1.845
Social functioning	2.1%	14.151	0.000 ***	−2.063				
Role limitation due to emotional problems	1.5%	10.118	0.002 **	−3.209				
Mental health	4.1%	27.395	0.000 ***	−3.670	0.7%	4.998	0.026 *	−1.575

SF-36: 36-item Short Form Health Survey. * *p* < 0.05 (two-tailed). ** *p* < 0.01 (two-tailed). *** *p* < 0.001 (two-tailed).

## Data Availability

The data presented in this study are available upon reasonable request from the corresponding author. The data are not publicly available as they contain information that could compromise the privacy matters of the early adolescents who participated in the study.
